# Effect of *Lactobacillus rhamnosus* Probiotic in Early Pregnancy on Plasma Conjugated Bile Acids in a Randomised Controlled Trial

**DOI:** 10.3390/nu13010209

**Published:** 2021-01-13

**Authors:** Yutao Chen, Jun Lu, Kristin Wickens, Thorsten Stanley, Robyn Maude, Peter Stone, Christine Barthow, Julian Crane, Edwin A. Mitchell, Fabrice Merien, Rinki Murphy

**Affiliations:** 1 College of Life and Marine Sciences, Shenzhen University, Shenzhen 518071, China; yutao.chen@aut.ac.nz; 2 Faculty of Health and Environmental Sciences, School of Science, Auckland University of Technology, Auckland 1142, New Zealand; fabrice.merien@aut.ac.nz; 3 Faculty of Health and Environmental Sciences, School of Public Health and Interdisciplinary Studies, Auckland University of Technology, Auckland 0627, New Zealand; 4 Institute of Biomedical Technology, Auckland University of Technology, Auckland 1142, New Zealand; 5 Maurice Wilkins Centre for Molecular Biodiscovery, Auckland 1142, New Zealand; 6 Departments of Medicine and Paediatrics, University of Otago Wellington, Wellington 6021, New Zealand; kristin.wickens@otago.ac.nz (K.W.); christine.barthow@otago.ac.nz (T.S.); thorsten.stanley@otago.ac.nz (C.B.); julian.crane@otago.ac.nz (J.C.); 7 School of Nursing, Midwifery, and Health Practice, Wellington Faculty of Health, Victoria University of Wellington, Wellington 6140, New Zealand; robyn.maude@vuw.ac.nz; 8 School of Medicine, Faculty of Medical and Health Sciences, University of Auckland, Auckland 1142, New Zealand; p.stone@auckland.ac.nz (P.S.); e.mitchell@auckland.ac.nz (E.A.M.); 9 Auckland Diabetes Centre, Auckland District Health Board, Auckland 1142, New Zealand; 10 Whitiora Diabetes Department, Counties Manukau District Health Board, Auckland 1640, New Zealand

**Keywords:** probiotics, gestational diabetes, bile acids, insulin sensitivity, randomised controlled trial, conjugated bile acids, LC-MS

## Abstract

We have previously shown that probiotic supplementation with *Lactobacillus rhamnosus* HN001 (HN001) led to a reduced incidence of gestational diabetes mellitus (GDM). Here we investigate whether HN001 supplementation resulted in alterations in fasting lipids, insulin resistance, or bile acids (BAs) during pregnancy. Fasting plasma samples collected at 24–30 weeks’ gestation, from 348 women randomised at 14–16 weeks’ gestation to consume daily probiotic HN001 (*n* = 172) or a placebo (*n* = 176) were analysed for lipids, insulin, glucose and BAs. Women supplemented with HN001 had lower fasting glucose compared with placebo (*p* = 0.040), and lower GDM. Significant differences were found in fasting insulin, HOMA-IR, low density lipoprotein-cholesterol (LDL-c), high density lipoprotein (HDL)-c, triglycerides, total cholesterol, and BAs by GDM status. Lower fasting conjugated BAs were seen in women receiving HN001. A significant decrease of glycocholic acid (GCA) was found in older (age ≥ 35) women who received HN001 (*p* = 0.005), while GDM women showed significant reduced taurodeoxycholic acid (TDCA) (*p* = 0.018). Fasting conjugated BA was positively correlated with fasting glucose (*r* = 0.136, *p* = 0.020) and fasting insulin (*r* = 0.113, *p* = 0.036). Probiotic HN001 supplementation decreases conjugated BAs and might play a role in the improvement of glucose metabolism in women with pregnancy.

## 1. Introduction

Gestational diabetes mellitus (GDM) is increasing in prevalence, and carries increased morbidity for both mother and child [1]. GDM increases the risk of mothers developing pre-eclampsia, preterm birth, induction of labour and caesarean section [1], while babies exposed to hyperglycaemia in utero are at risk of fetal death, macrosomia, birth trauma, hyaline membrane disease, and neonatal hypoglycaemia [2]. In addition, GDM increases the risk of later obesity and type 2 diabetes in both the mother and her offspring. GDM is associated with increased maternal insulin resistance of pregnancy that cannot be met by a sufficient pancreatic insulin response to lower blood glucose to normal levels. Insulin resistance is commonly associated with dyslipidaemia, characterised by elevated triglycerides, low-density lipoprotein (LDL) cholesterol and lowered high-density lipoprotein (HDL) cholesterol.

The potential role of gut microbiota in reducing insulin resistance, as well as improving lipid profiles, has resulted in an interest in using certain probiotic bacteria in the prevention of GDM [2]. Probiotics are viable microorganisms that can benefit the health of their host when ingested in adequate quantities [3]. Jafarnejad et al. showed that intake of the probiotic mixture (VSL #3) containing eight strains of lactic acid bacteria (*Streptococcus thermophilus, Bifidobacterium breve, Bifidobacterium longum, Bifidobacterium infantis, Lactobacillus acidophilus, Lactobacillus plantarum, Lactobacillus paracasei,* and *Lactobacillus delbrueckii* subsp. *Bulgaricus*) in women with GDM at 16 weeks of gestation significantly reduced the magnitude of insulin resistance in comparison with the placebo group after 8 weeks of supplementation [4]. Karamali et al. reported improved glycaemic control and significant decreases in triglycerides and VLDL cholesterol concentrations in GDM women at 24–28 weeks of gestation after six-week consumption of probiotic capsule containing *L. acidophilus*, *L. casei* and *B. bifidum* strains [5]. We previously reported that supplementing pregnant mothers with the probiotic *Lactobacillus rhamnosus* HN001 (HN001) lowered the prevalence of GDM, particularly in women aged ≥ 35 years (*p* = 0.009) and those previously diagnosed with GDM (*p* = 0.004) [6]. It is not known whether HN001 also causes improvements in lipids or insulin sensitivity during pregnancy.

One of the ways that probiotics could exert beneficial effects on glucose metabolism, lipids and insulin resistance is by altering the gut microbial metabolism of bile acids (BA). BAs are endocrine molecules that regulate numerous metabolic processes, including glucose, lipid, and energy homeostasis [7], and are associated with pregnancy-related diseases in the second and third trimesters, including intrahepatic cholestasis of pregnancy (ICP), GDM, and gestational hyperlipidaemia [8,9]. The primary BAs cholic acid (CA) and chenodeoxycholic acid (CDCA) are synthesised from cholesterol in the liver, which is conjugated to either glycine or taurine before secretion into the bile. Bacterial action in the intestine leads to modification from the primary BA by deconjugation, dehydroxylation, dehydrogenation, and epimerisation. This generates secondary BA, which includes deoxycholic acid (DCA), ursodeoxycholic acid (UDCA), lithocholic acid (LCA), and hyodeoxycholic acid (HDCA). Secondary BA can then be re-conjugated with taurine or glycine [10].

BA sequestrant treatments have been shown to be effective for type 2 diabetes and hypercholesterolaemia. However, the data on the individual circulating BA’s relationship with metabolic improvements are less clear. Higher plasma levels of total BA [11] and conjugated BA levels (GHDCA and THDCA) [12] have been observed among women with GDM when compared to healthy controls. Higher glycine-conjugated (GDCA, GCA, and GCDCA) and taurine-conjugated (TCDCA and TCA) BAs have also been observed in adults with T2DM, and were associated with lower insulin sensitivity and higher plasma triglyceride levels [13]. DCA is less consistent as one study showed lower levels of DCA and GUDCA in early pregnancy was associated with later GDM in Chinese women [14]. Another study showed that higher levels of DCA along with improved insulin sensitivity was observed following the intake of the probiotic *Lactobacillus reuteri* DSM 17,938 among men and women with T2DM [15]. No studies have evaluated the effect of probiotic supplementation with HN001 during pregnancy on plasma BAs, or possible alterations in glucose metabolism or lipid profiles in association with GDM.

We hypothesised that maternal probiotic supplementation would alter gut microbiota function by altering secondary bile acids which would lower fasting lipid profiles and insulin resistance in pregnant women. Hence, we analysed the relationships between BAs, lipids, and insulin resistance in relation to probiotic supplementation in women who took part in a randomised, double blind, placebo-controlled trial of HN001 in early pregnancy. Since gut microbiota function differs by age, obesity and GDM status, and our previous study showed a beneficial impact of HN001 on lowering the incidence of GDM among women with older age, and previous GDM, we stratified our results by these factors.

## 2. Materials and Methods

### 2.1. Study Design

The study has previously been described in detail [16]. In brief, it was a two-centre (Wellington and Auckland), double-blind, randomised, placebo-controlled trial investigating the effects of probiotic supplementation in early pregnancy on subsequent infant development of eczema (primary outcome) and maternal GDM (secondary outcome).

### 2.2. Participants

A total of 432 pregnant women in Auckland and Wellington, New Zealand, were recruited between 14–16 weeks’ gestation. In order to enrich for infant eczema and allergy outcomes, only pregnant women who themselves or their unborn child’s biological father had a personal history of asthma, hay fever, or eczema requiring medication, were recruited. The study received ethical approval from the New Zealand Multi-Region Ethics Committee (MEC/11/09/077). Trial registration: Australian New Zealand Clinical Trials Registration (ACTRN12612000196842).

### 2.3. Intervention

**Figure 1:**
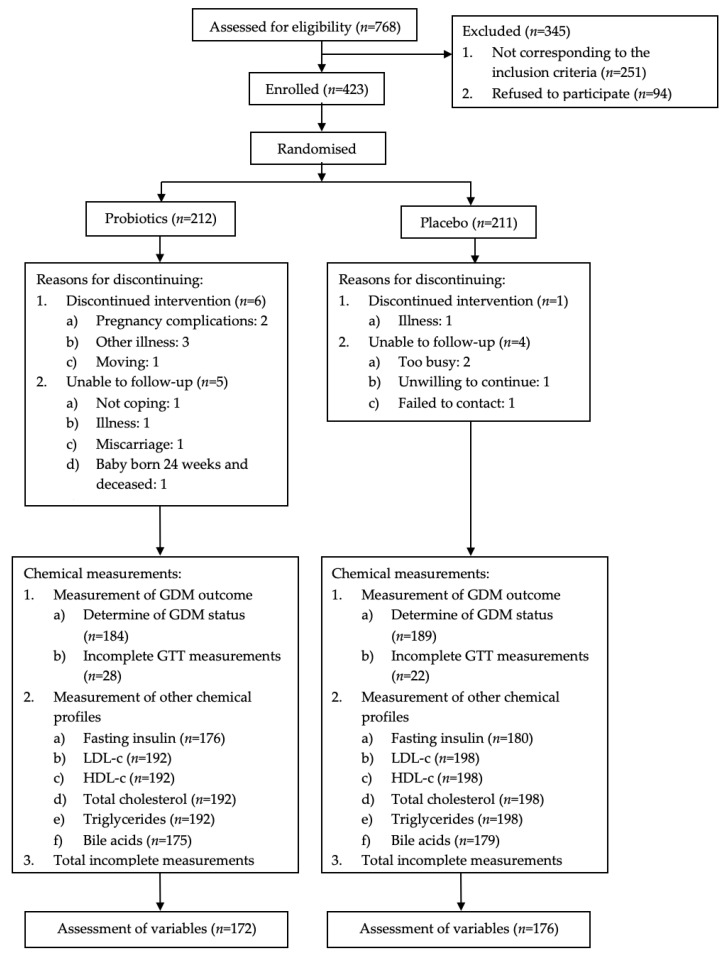
Design of the study, status of study participants and data collections through the trial. GDM = gestational diabetes mellitus, GTT = glucose tolerance test, LDL-c = low-density lipoprotein-cholesterol, HDL-c = high-density lipoprotein-cholesterol.

The pregnant women were randomised to consume a tablet containing either 6×10^9^ colony-forming units (CFU) daily of *Lactobacillus rhamnosus* HN001 or a placebo (both supplied by Fonterra Co-operative Group Ltd., Auckland, New Zealand). Participants by study centres were stratified according to a computer-generated randomisation schedule and an allocation ratio of 1:1 and randomised to HN001 or a placebo in blocks of 20 by a Fonterra staff member. All researchers, relevant staff, and participants were blinded to study treatment allocation. An overview of the study design is shown in Figure 1.

### 2.4. Data Collection

The age, weight (kg), waist circumference (cm), and body mass index (BMI) (kg m^−2^) of the participants were recorded at 14 to 16 weeks’ gestation. The oral glucose tolerance test (OGTT) and the evaluation of plasma glucose were conducted at a community laboratory among studied participants at 24 to 30 weeks’ gestation. Additional plasma samples collected during fasting were immediately centrifuged when samples arrived at the laboratory and subsequently stored as aliquots at −80 °C until analysis. The stored plasma samples from 24 to 30 weeks’ gestation were used for biochemical analysis in this study. The plasma concentrations of glucose, insulin, low-density lipoprotein-cholesterol (LDL-c), high-density lipoprotein-cholesterol (HDL-c), total cholesterol, and triglycerides were measured in the fasting state by an auto-analyser (Roche Diagnostics, Basel, Switzerland) according to the manufacturer’s protocols. BAs were measured using an established liquid chromatography-tandem mass spectrometry (LC-MS/MS) method described previously [17] with a slight modification to optimise the detection sensitivity suggested by a previous publication [18]. The LC-MS/MS system consisted of an HPLC Agilent 1200 series apparatus and the Agilent 6420 Triple Quadrupole MS/MS (Agilent Technologies, Santa Clara, CA, USA). The fasting BA analysis included CA, cholic acid; CDCA, chenodeoxycholic acid; DCA, deoxycholic acid; UDCA, ursodeoxycholic acid; GCA, glycocholic acid; GCDCA, glycochenodeoxycholic acid; GDCA, glycodeoxycholic acid; GUDCA, glycoursodeoxycholic acid; TCDCA, taurochenodeoxycholic acid; TDCA, taurodeoxycholic acid; TUDCA, tauroursodeoxycholic acid; TLCA, taurolithocholic acid; and THDCA, taurohyodeoxycholic acid. Due to undetectable plasma concentrations, the records of lithocholic acid (LCA) and hyodeoxycholic acid (HDCA) were removed from all calculations and analyses.

### 2.5. Definitions

GDM status was determined based on either the IADPSG recommendations (fasting plasma glucose ≥5.1 mmol L^−1^, or 1-h glucose ≥10 mmol L^−1^, or 2-h glucose ≥8.5 mmol L^−1^) [19] or on the NZ definition of GDM (fasting plasma glucose ≥5.5 mmol L^-1^ or 2-h glucose ≥9 mmol L^−1^) [20]. The definition of obesity was based on the National Institutes of Health (NIH)’s guideline, which suggested a BMI of 30 kg m^−2^ and above. Insulin resistance was estimated using the homeostatic model assessment of insulin resistance (HOMA-IR).

BA compositions were classified according to their site of synthesis (primary vs. secondary) or conjugation state (unconjugated vs. conjugated). The molar sum of BA concentrations in each category was used to determine the levels of each BA composition. Compositions included: (1) total BA = all 13 BAs; (2) primary BA = CA, GCA, CDCA, GCDCA, and TCDCA; (3) secondary BA = DCA, GDCA, TDCA, UDCA, GUDCA, TUDCA, TLCA, and THDCA; (4) unconjugated BA = all unconjugated BAs; (5) conjugated BA = all glycine and taurine conjugated BAs; (6) glycine-conjugated BA; (7) Taurine-conjugated BA; (8) primary-unconjugated BA; (9) primary-conjugated BA; (10) secondary-unconjugated BA; and (11) secondary-conjugated BA.

### 2.6. Statistical Analysis

Generated data were analysed using GraphPad Prism version 8.4.3 (Graphpad Software, San Diego, CA, USA). Normal distribution of model residuals was tested with the Kolmogorov–Smirnov test or the Shapiro–Wilk test, as appropriate. Significant differences between groups were evaluated using unpaired student’s t-tests, the Mann–Whitney U test or the Chi-square test. Data were presented as mean ±SD, number (%) or median (IQR) as required. The correlation assays were performed using the Spearman’s rank test. Statistical significance was set at *p* < 0.05 (two-tailed). The pregnant women were stratified according to key characteristics that were associated with the beneficial impact of probiotics: age ≥35 vs. age <35; prior GDM vs. non-GDM; and obesity (BMI ≥ 30 kg m-2) vs. non-obesity (BMI < 30 kg m^−2^), as previously described [21]. The stratification was followed by the analysis of variations in chemical profiles between the probiotic supplementation group (HN001) and the placebo group (Placebo). Table 1 shows all of the subsets studied within this trial.

## 3. Results

### 3.1. Characteristics of the Study Population

Of the total number of participants (*n* = 423) who were randomised to the HN001 (*n* = 212) or placebo group (*n* = 211), 184 (87%) participants in the HN001 group and 189 (90%) participants in the placebo group completed the weeks 24–30 OGTT results, which contained all three time points (fasting, 1 h and 2 h), at 27.7 ± 4.6 and 28.0 ± 8.6 weeks’ gestation, respectively. Incomplete biochemical assessments (HN001 vs. Placebo, 40 (19%) vs. 35 (17%)) were either due to discontinued intervention, loss to follow-up, insufficient aliquoting of the samples, or other unexpected failures during the analysis. In total, the data for 172 (81%) and 176 (83%) participants from the HN001 group and the placebo group, respectively, were included for the assessment of additional biochemical indices (insulin, lipids, BAs) (Figure 1). Table 2 shows the baseline characteristics of the 348 studied participants. There were no significant differences between the HN001 vs. placebo groups at baseline concerning parameters of age, weight, waist circumference, BMI, or ethnicity.

### 3.2. Effect of L. rhamnosus HN001 Supplementation on GDM, Glucose, HOMA-IR, and Lipid Profiles

As shown in Table 2, we observed a significantly lower GDM prevalence in the HN001 group compared with the placebo group, consistent with the results from the larger group [5]: by IADPSG definition, HN001 vs. Placebo, 13 (7.6%) vs. 25 (14.2%), *p* = 0.047; by NZ definition, HN001 vs. Placebo, 3 (1.7%) vs. 10 (5.7%), *p* = 0.053) and a significant decrease in fasting glucose (HN001 vs. Placebo, 4.3 (4.1–4.5) vs. 4.4 (4.1–4.6) mmol L^-1^, *p* = 0.040). Since the number of GDM individuals by NZ definition in the HN001 group (*n* = 3) was insufficient for conducting further stratification, only the IADPSG definition of GDM was considered for the following subset analyses.

We did not observe any significant effect of HN001 supplementation on fasting insulin (although the mean of HN001 group is higher than that of placebo), HOMA-IR, lipids (LDL-c, HDL-c, total cholesterol, and triglycerides) among all 348 studied participants, even when stratified by GDM (Table 3), maternal age, or obesity status (data not shown).

### 3.3. Effect of L. rhamnosus HN001 Supplementation on BAs

**Figure 2:**
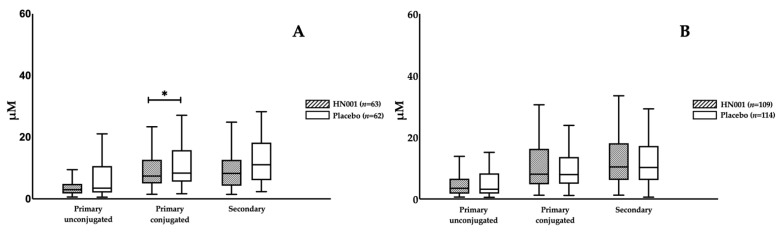
Fasting levels of bile acid compositions in the participants supplemented with *L. rhamnosus* HN001 or placebo stratified by age groups. (**A**) shows the subset of older women (Age ≥ 35 years, *n* = 125), while (**B**) shows the subset of young women (Age < 35 years, *n* = 223). Data are presented as median and interquartile range. * *p* < 0.05.

**Figure 3:**
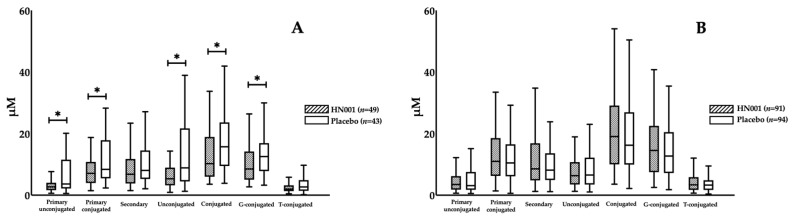
Fasting levels of bile acid compositions in the participants supplemented with *L. rhamnosus* HN001 or placebo stratified by age groups and obesity status. (**A**) shows the subset of non-obese older women (Age ≥ 35 and non-obese, *n* = 92), while (**B**) shows the subset of non-obese young women (Age < 35 and non-obese, *n* = 185). Data are presented as median and interquartile range. * *p* < 0.05.

**Figure 4:**
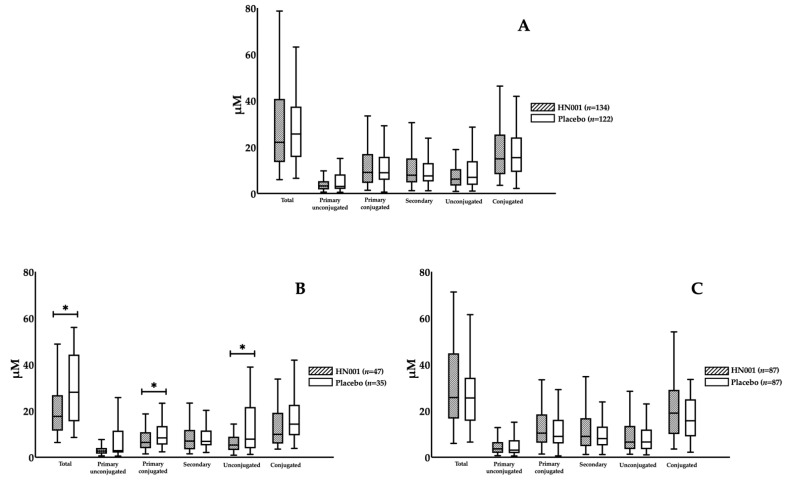
Fasting levels of bile acid compositions in the participants supplemented with *L. rhamnosus* HN001 or placebo stratified by age groups, IADPSG GDM and obesity status. (**A**) shows the subset of non-obese women without GDM (non-GDM and non-obese, *n* = 256), while (**B**) shows the subset of non-obese older women without GDM (Age ≥ 35 and non-GDM and non-obese, *n* = 82), and (**C**) presents the subset of non-obese young women without GDM (Age < 35 and non-GDM and non-obese, *n* = 174). Data are presented as median and interquartile range. * *p* < 0.05.

**Figure 5:**
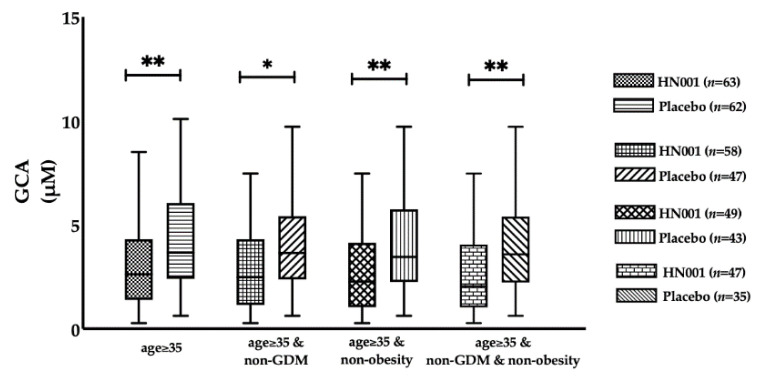
Fasting levels of glycocholic acid (GCA) in the participants supplemented with *L. rhamnosus* HN001 or placebo stratified by age groups, IADPSG GDM and obesity status. Data are presented as median and interquartile range. * *p* < 0.05, ** *p* < 0.01.

There were no changes in any BAs as a consequence of HN001 treatment among all participants (Table 2). However, we noticed that probiotic impacts on BAs differed by maternal age, in which HN001 significantly lowered the fasting levels of primary conjugated BA compared with the placebo group (8.25 (4.25–12.49) vs. 11.07 (6.24–18.05) μM, HN001 vs. placebo, *p* = 0.029) in the older women (Figure 2A), while no differences in BA composition were found in the young women (Figure 2B). When further stratified by BMI, we found that primary conjugated BA significantly decreased with HN001 supplementation among slim, older women (7.08 (3.98–10.73) vs. 8.37 (5.68–15.68) μM, *p* = 0.017) (Figure 3A). This lowering of primary conjugated BA was seen in women without prior GDM (6.41 (3.95–10.80) vs. 8.37 (5.68–13.38) μM, *p* = 0.026) (Figure 4B). Total BA composition was significantly lower among slim, older women without GDM, who received HN001 (17.66 (11.53–25.69) vs. 28.09 (16.41–42.80) μM, *p* = 0.017) (Figure 4B). HN001 intervention resulted in lower primary conjugated BA among older women without GDM, but the difference was not significant compared with the placebo group (data not shown). What was common among the above subgroups, which focused particularly on older individuals, was that HN001 supplementation significantly decreased the GCA levels (Figure 5).

**Figure 6:**
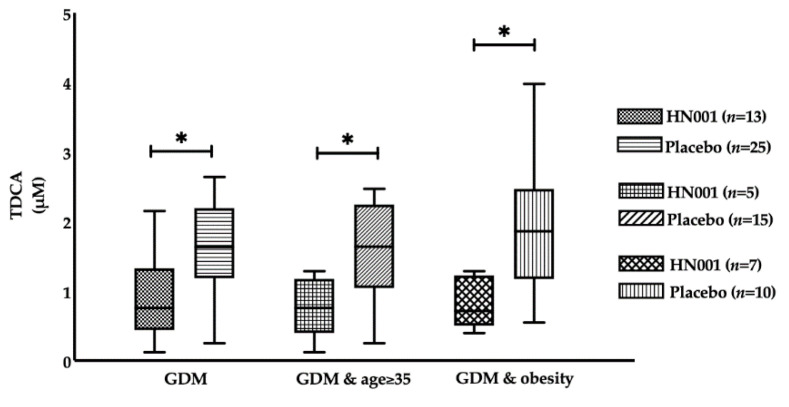
Fasting levels of taurodeoxycholic acid (TDCA) in the participants supplemented with *L. rhamnosus* HN001 or placebo stratified by age groups, IADPSG GDM and obesity status. Data are presented as median and interquartile range. * *p* < 0.05.

Among the subset with GDM, those who were supplemented with HN001 had significantly lower TDCA compared to those receiving a placebo (0.76 (0.48–1.29) vs. 1.63 (1.19–2.20) μM, *p* = 0.018) (Table 3). Under further stratification, TDCA also significantly decreased in the HN001 group among older women with GDM (0.71 (0.60–1.16) vs. 1.86 (1.25–2.34) μM, *p* = 0.015) and obese women with GDM (0.76 (0.40–1.17) vs. 1.63 (1.21–2.13) μM, *p* = 0.025) (Figure 6).

### 3.4. Correlations between Metabolic Profiles and Bile Acids

The associations between the metabolic parameters and selected BA individuals (as well as classified compositions) are shown in Table 4. Total BA was positively correlated with fasting glucose (r = 0.136, *p* = 0.011). Conjugated BA was positively correlated with fasting glucose (r = 0.125, *p* = 0.020) and fasting insulin (r = 0.113, *p* = 0.036). Additionally, G-conjugated BA was also positively correlated with fasting insulin (r = 0.105, *p* = 0.049), while T-conjugated BA was positively associated with 1-h postprandial glucose (r = 0.125, *p* = 0.020). In terms of BA individuals, GCA was positively associated with fasting insulin (r = 0.147, *p* = 0.006) and HOMA-IR (r = 0.132, *p* = 0.014). GUDCA was positively correlated with triglyceride (r = 0.141, *p* = 0.008). No significant correlations were seen among TDCA and other metabolic profiles.

## 4. Discussion

There was no overall significant impact of HN001 probiotic supplementation on HOMA-IR insulin resistance, lipids or BA measured at 24–30 weeks’ gestation. Among women over the age of 35 years, those who received HN001 had lower fasting levels of primary conjugated BA compared with the placebo group. Since this was a small subset (5 vs. 15), this finding requires validation in larger studies. Conjugated BA was positively correlated with fasting glucose and insulin.

Although this [6] and other studies [4,22] have demonstrated that probiotic supplementation during pregnancy has beneficial effects on GDM, probiotic interventions have shown inconsistent effects on fasting insulin or HOMA-IR in pregnancy. Asemi et al. investigated the effect of daily consumption of probiotic-supplemented yoghurt containing multiple probiotic species, including *Lactobacillus* and *Bifidobacterium* strains, among 70 pregnant women without GDM in their third trimester. This showed no effect on the serum insulin levels and the HOMA-IR score [23]. However, 9 weeks of probiotic supplementation elicited significant differences in both serum insulin (+ 1.2 ± 1.2 vs. +5.0 ± 1.1 *μ*IU/mL, probiotic vs. placebo, *p* = 0.02) and insulin resistance (−0.2 ± 0.3 vs. +0.7 ± 0.2 *μ*IU/mL, *p* = 0.01) from the baseline [23]. On the other hand, in another clinical trial, 8 weeks supplementation using a VSL#3 probiotic capsule containing eight strains of lactic acid bacteria among 82 GDM women at 16 weeks of gestation significantly lowered insulin levels (16.6 ± 5.9 vs. 22.3 ± 4.9 *μ*IU/mL, *p* = 0.04) and lowered HOMA-IR (3.7 ± 1.5 vs. 4.9 ± 1.2 *μ*IU/mL, *p* = 0.03). However, when compared to the within-group differences from the baseline, insulin levels and HOMA-IR remained unchanged in the probiotic and placebo group [4]. Another study among normoglycaemic participants who received dietary counselling showed that probiotic supplementation with *Lactobacillus rhamnosus* GG and *Bifidobacterium lactis* Bb12 lowered insulin resistance, as estimated by HOMA-IR, in the third trimester of pregnancy (1.49 (95%CI 1.31, 1.71) vs. 1.90 (95%CI 1.66, 2.17), *p* = 0.040). At the same time, no significant improvement effect was found in insulin levels [24].

Previous clinical trials to assess the effect of probiotic supplementation on lipid profiles also produced conflicting results. In a study among GDM or impaired glucose-tolerant pregnant women (<34 weeks’ gestation), daily *Lactobacillus salivarius* UCC118 supplementation for 4–6 weeks (from diagnosis until delivery) was found to lower cholesterol concentrations, particularly LDL, after adjusting for their baseline values, compared with the placebo groups [25]. Karamali et al. reported that after 6 weeks of intervention, a significant decrease in serum triglycerides from baseline was noted (−1.6 ± 59.4 vs. +27.1 ± 37.9 mg/dL, *p* = 0.03) among the group of participants at 24–28 weeks gestation who took a daily probiotic capsule that contained various bacterial species and strains [5]. Nevertheless, no significant within- or between-group differences in total, HDL or LDL cholesterol, were noticed [5]. Additionally, a study conducted by Hoppu et al. investigated the influence of dietary counselling versus probiotic administration of a capsule containing a mixture of *Lactobacillus* and *Bifidobacterium* species in the first trimester of pregnancy; in this population of 256 healthy women, no between-group differences in lipid levels were found in the third trimester [26].

Overall, the discrepant findings could be the result of differences in probiotic species or strains, dosage, maternal age, BMI, GDM status and gestation of the recruited participants. Based upon a meta-analysis, a dose of more than 10^7^ CFUs could show the beneficial effects of probiotic supplementation on the metabolic health of pregnant women [27]. Furthermore, a dosage ranging from 10^8^ to 10^10^ CFU/d was suggested to be sufficient to cause effective metabolic changes [28]. In the current study, 6 × 10^9^ CFU was adequate in reducing GDM and mean glycaemia; however, it did not have a discernible impact on insulin or lipid profiles. This strain may therefore have a direct effect on GDM without impacting circulating insulin or lipid levels.

Maternal gut microbiota composition and function may differ depending on the age [29], BMI [30], and GDM status [31] of the host. Correspondingly, the impact of probiotic supplementation during pregnancy may vary among individuals due to the distinct interactions between the given probiotic and intestinal bacteria, which may result in different physiological or immune responses [32]. Therefore, we analysed whether the impact of HN001 on metabolic parameters in pregnant women differed when stratified by different criteria.

Our data demonstrated that the probiotic HN001 at a dose of 6 × 10^9^ CFU/d lowers taurine-conjugated BAs (mainly TDCA) among the GDM women. GCA was positively correlated with fasting insulin and HOMA-IR, which was also decreased by HN001 among older participants. These observations are consistent with conjugated BAs leading to impaired glycaemic control during pregnancy, which were decreased by probiotics and thereby contributing to favourable maternal glucose metabolism. We also found that total fasting BA was significantly reduced under HN001 intervention in lean, older women without GDM. Kong et al. indicated that high levels of maternal BA circulating at 14–18 gestational weeks were significantly associated with the risk of GDM [11], which was in line with our finding that total BA was positively associated with fasting glucose.

We found that glycine-conjugated BA was positively associated with fasting insulin and HOMA-IR. This finding is consistent with a recent study that reported altered BA metabolism among GDM women, in which a positive correlation between fasting, insulin, and HOMA-IR and glycine- and taurine-conjugated BAs was observed [33]. THDCA value was higher in mothers with GDM [12], though such a difference was not seen in our study. However, we observed that THDCA was positively associated with insulin and HOMA-IR. In addition, we observed significant positive correlations between TCDCA and fasting glucose, along with 1-hour postprandial glucose. Our observation is in line with a previous report suggesting that taurine-conjugated BAs were positively correlated with fasting glucose, post-load glucose, fasting insulin, and HOMA-IR [34].

Our previous study reported the beneficial impact of HN001 on lowering the rate of GDM among women with older age, and previous GDM. However, this effect was not significant when stratifying by BMI [6]. Although no metabolic improvement under HN001 intervention in either stratified groups were seen in this study, we observed that the HN001 impact on altering plasma BAs was different between individuals with a BMI ≥ 30 kg m^−2^ and those with a BMI < 30 kg m^−2^. Culpepper et al. reported that probiotic supplementation using a mixture of *Bacillus subtilis* and *Bifidobacterium lactis* increased the deconjugation of plasma BAs in individuals with a BMI ≥ 30 kg m^−2^ but this had no discernible effect on glucose metabolism or serum cholesterol [35].

## 5. Conclusions

In conclusion, *Lactobacillus rhamnosus* HN001 supplementation during pregnancy appears to lower conjugated BAs, which might play a role in improving glucose metabolism, but this does not appear to have a significant effect on fasting lipids. Since the bile acid lowering effects of HN001 were greatest among leaner, older women, further studies evaluating its impact on GDM prevention in this subgroup of women is warranted.

## Figures and Tables

**Table 1:** Stratification of the participants.

Subset Titles	Definitions	Number of Participants (*n*)		
		HN001	Placebo	Total
Age ≥ 35 years	Older women	63	62	125
Age < 35 years	Young women	109	114	223
GDM	Women with GDM	13	25	38
Age ≥ 35 and GDM	Older women with GDM	5	15	20
Age ≥ 35 and non-GDM	Older women without GDM	58	47	105
Age < 35 and non-GDM	Young women without GDM	101	104	205
Age ≥ 35 and non-obese	Non-obese older women	49	43	92
Age < 35 and non-obese	Non-obese young women	91	94	185
non-GDM and non-obese	Non-obese women without GDM	134	122	256
Age ≥ 35 and non-GDM and non-obese	Non-obese older women without GDM	47	35	82
Age < 35 and non-GDM and non-obese	Non-obese young women without GDM	87	87	174
GDM and obese	Obese women with GDM	7	10	17

GDM = gestational diabetes mellitus.

**Table 2:** Clinical characteristics, ethnicities, fasting metabolic indices and bile acid profiles of the participants supplemented with *L. rhamnosus* HN001 or placebo.

	HN001 (*n* = 172)	Placebo (*n* = 176)	*p*-Value
Baseline Characteristics			
Age (years)	33.1 ± 4.2	33.8 ± 4.3	0.144
Weight (kg)	68.4 (63.0–79.1)	71.1 (63.3–81.9)	0.194
Waist circumference (cm)	86.4 (79.8–93.8)	86.8 (80.6–99.1)	0.146
BMI (kg m^−2^)	25.1 (22.9–28.6)	25.8 (23.0–30.0)	0.209
Obesity statistics	32 (18.6%)	39 (22.2%)	0.412
Diagnosed GDM (IADPSG)	13 (7.6%)	25 (14.2%)	0.047
Diagnosed GDM (NZ definition)	3 (1.7%)	10 (5.7%)	0.053
Fasting metabolic variables			
Fasting glucose (mmol L^−1^)	4.3 (4.1–4.5)	4.4 (4.1–4.6)	0.040
1-h glucose (mmol L^−1^)	6.6 (5.6–7.7)	6.7 (5.7–8.1)	0.258
2-h glucose (mmol L^−1^)	5.5 (4.9–6.3)	5.5 (4.7–6.5)	0.791
Insulin (pmol L^−1^)	64.37 (48.58–92.43)	60.09 (41.93–86.00)	0.134
HOMA-IR	1.74 (1.24–2.49)	1.64 (1.12–2.45)	0.363
LDL-c (mmol L^−1^)	3.76 (3.20–4.57)	3.66 (3.00–4.56)	0.287
HDL-c (mmol L^−1^)	1.92 (1.60–2.19)	1.93 (1.69–2.18)	0.450
Total cholesterol (mmol L^−1^)	6.26 (5.50–7.05)	6.11 (5.33–6.98)	0.426
Triglycerides (mmol L^−1^)	1.72 (1.34–2.07)	1.61 (1.30–1.98)	0.264
Fasting bile acids			
CA (μM)	1.86 (0.77–4.23)	1.61 (0.84–7.04)	0.511
CDCA (μM)	1.10 (0.50–1.91)	1.24 (0.63–1.89)	0.413
GCA (μM)	3.41 (1.62–5.63)	3.48 (2.24–6.06)	0.243
GCDCA (μM)	4.24 (2.26–7.44)	4.39 (2.70–7.89)	0.433
TCDCA (μM)	1.29 (0.69–2.63)	1.64 (0.76–3.10)	0.133
DCA (μM)	1.03 (0.52–1.94)	1.00 (0.51–1.78)	0.981
UDCA (μM)	0.93 (0.47–1.71)	0.82 (0.41–1.76)	0.366
GDCA (μM)	3.07 (1.37–6.09)	3.18 (1.74–5.77)	0.488
GUDCA (μM)	0.36 (0.19–0.75)	0.43 (0.22–0.76)	0.480
TDCA (μM)	1.13 (0.50–1.95)	1.15 (0.55–1.87)	0.759
TUDCA (μM)	0.06 (0.02–0.12)	0.05 (0.03–0.10)	0.907
TLCA (μM)	0.06 (0.04–0.09)	0.06 (0.04–0.10)	0.459
THDCA (μM)	0.03 (0.01–0.07)	0.03 (0.01–0.06)	0.662

BMI = body mass index, HOMA-IR = homeostatic model assessment of insulin resistance, LDL-c = low-density lipoprotein-cholesterol, HDL-c = high-density lipoprotein-cholesterol, CA = cholic acid, CDCA = chenodeoxycholic acid, GCA = glycocholic acid, GCDCA = glycochenodeoxycholic acid, TCDCA = taurochenodeoxycholic acid, DCA = deoxycholic acid, UDCA = ursodeoxycholic acid, GDCA = glycodeoxycholic acid, GUDCA = glycoursodeoxycholic acid, TDCA = taurodeoxycholic acid, TUDCA = tauroursodeoxycholic acid, TLCA = taurolithocholic acid, THDCA = taurohyodeoxycholic acid.

**Table 3:** Fasting metabolic indices and bile acid profiles of the participants supplemented with *L. rhamnosus* HN001 or placebo by IADPSG definition of GDM.

	non-GDM			GDM		
	HN001 (*n* = 159)	Placebo (*n* = 151)	*p*-Value	HN001 (*n* = 13)	Placebo (*n* = 25)	*p*-Value
Fasting metabolic variables						
Fasting glucose (mmol L^−1^)	4.2 (4.1–4.5)	4.3 (4.1–4.5)	0.186	4.9 (4.2–5.1)	4.8 (4.5–5.3)	0.536
1-h glucose (mmol L^−1^)	6.5 (5.5–7.4)	6.4 (5.6–7.5)	0.949	10.5 (10.0–10.7)	10.0 (8.9–10.5)	0.355
2-h glucose (mmol L^−1^)	5.5 (4.8–6.1)	5.4 (4.7–6.1)	0.699	7.0 (5.7–8.8)	7.0 (6.5–8.8)	0.961
Insulin (pmol L^−1^)	62.87 (47.71–84.66)	55.86 (39.57–79.61)	0.046	104.90 (90.32–183.80)	84.62 (64.45–136.90)	0.234
HOMA-IR	1.70 (1.18–2.29)	1.47 (1.06–2.29)	0.092	3.27 (1.92–5.27)	2.81 (1.79–4.29)	0.259
LDL-c (mmol L^−1^)	3.76 (3.25–4.57)	3.67 (3.07–4.57)	0.407	3.65 (2.94–4.56)	3.61 (2.50–4.40)	0.494
HDL-c (mmol L^−1^)	1.92 (1.6–2.20)	1.93 (1.70–2.18)	0.348	1.80 (1.55–2.06)	1.78 (1.39–2.30)	0.988
Total cholesterol (mmol L^−1^)	6.28 (5.52–7.06)	6.12 (5.43–6.97)	0.602	6.10 (5.50–6.88)	6.03 (5.12–7.07)	0.459
Triglycerides (mmol L^−1^)	1.66 (1.33–2.07)	1.55 (1.30–1.96)	0.157	1.95 (1.79–2.07)	1.94 (1.39–2.43)	0.896
Fasting bile acids						
CA (μM)	1.94 (0.80–4.31)	1.52 (0.83–7.07)	0.756	1.48 (0.71–3.38)	2.63 (1.08–4.21)	0.361
CDCA (μM)	1.15 (0.53–1.89)	1.25 (0.61–1.78)	0.678	0.58 (0.32–2.31)	1.11 (0.69–2.70)	0.188
GCA (μM)	3.42 (1.62–5.73)	3.43 (2.21–5.83)	0.470	3.34 (1.76–4.23)	3.78 (2.51–9.08)	0.210
GCDCA (μM)	4.27 (2.27–7.62)	4.35 (2.67–7.66)	0.697	3.86 (2.24–5.80)	4.73 (3.03–11.82)	0.314
TCDCA (μM)	1.29 (0.69–2.67)	1.61 (0.74–3.00)	0.303	1.42 (1.04–2.21)	2.68 (0.96–5.05)	0.222
DCA (μM)	1.02 (0.52–2.01)	0.98 (0.52–1.74)	0.843	1.40 (0.53–1.70)	1.20 (0.51–2.08)	0.584
UDCA (μM)	0.93 (0.46–1.76)	0.82 (0.42–1.66)	0.363	1.09 (0.74–1.68)	0.98 (0.35–1.96)	1.000
GDCA (μM)	3.07 (1.37–6.33)	3.00 (1.52–5.58)	0.926	3.00 (1.36–3.70)	4.13 (3.14–6.84)	0.079
GUDCA (μM)	0.36 (0.19–0.74)	0.44 (0.19–0.76)	0.596	0.33 (0.31–0.83)	0.43 (0.29–0.62)	0.832
TDCA (μM)	1.14 (0.50–2.06)	1.05 (0.52–1.66)	0.623	0.76 (0.48–1.29)	1.63 (1.19–2.17)	0.018
TUDCA (μM)	0.06 (0.02–0.12)	0.05 (0.03–0.09)	0.918	0.06 (0.03–0.14)	0.07 (0.02–0.16)	0.879
TLCA (μM)	0.06 (0.04–0.09)	0.06 (0.03–0.10)	0.748	0.05 (0.03–0.08)	0.07 (0.05–0.10)	0.199
THDCA (μM)	0.03 (0.01–0.07)	0.03 (0.02–0.06)	0.435	0.02 (0.01–0.09)	0.04 (0.01–0.07)	0.345

HOMA-IR = homeostatic model assessment of insulin resistance, LDL-c = low-density lipoprotein-cholesterol, HDL-c = high-density lipoprotein-cholesterol, CA = cholic acid, CDCA = chenodeoxycholic acid, GCA = glycocholic acid, GCDCA = glycochenodeoxycholic acid, TCDCA = taurochenodeoxycholic acid, DCA = deoxycholic acid, UDCA = ursodeoxycholic acid, GDCA = glycodeoxycholic acid, GUDCA = glycoursodeoxycholic acid, TDCA = taurodeoxycholic acid, TUDCA = tauroursodeoxycholic acid, TLCA = taurolithocholic acid, THDCA = taurohyodeoxycholic acid.

**Table 4:** Correlations of fasting bile acids with metabolic parameters.

	Fasting Glucose	1-h Glucose	2-h Glucose	Fasting Insulin	HOMA-IR	LDL-c	HDL-c	Total Cholesterol	Triglycerides
Total	0.136 *	0.099	−0.037	0.102	0.102	−0.013	0.010	−0.016	0.066
Conjugated	0.125 *	0.079	0.031	0.113 *	0.089	−0.053	−0.040	−0.060	0.061
Primary conjugated	0.091	0.109 *	0.005	0.123 *	0.114 *	−0.096	−0.032	−0.103	0.035
GCA	0.088	0.085	0.021	0.147 **	0.132 *	−0.079	−0.048	−0.086	0.065
GCDCA	0.084	0.073	−0.027	0.080	0.080	−0.076	−0.008	−0.083	0.016
TCDCA	0.036	0.134 *	0.021	0.113 *	0.094	−0.091	−0.036	−0.092	0.006
Secondary conjugated	0.085	0.074	0.053	0.076	0.074	0.007	0.059	0.011	0.062
GUDCA	0.145 **	0.069	0.041	0.107 *	0.124 *	0.019	0.000	0.035	0.141 **
TDCA	0.027	0.100	0.051	0.066	0.057	−0.009	0.051	−0.008	−0.012
G-conjugated	0.103	0.088	0.014	0.105 *	0.104	−0.047	0.009	−0.050	0.050
T-conjugated	0.030	0.125 *	0.019	0.075	0.058	−0.058	0.012	−0.058	-0.025

* *p* < 0.05, ** *p* < 0.01. HOMA-IR = homeostatic model assessment of insulin resistance, LDL-c = low-density lipoprotein-cholesterol, HDL-c = high-density lipoprotein-cholesterol, GCA = glycocholic acid, GCDCA = glycochenodeoxycholic acid, TCDCA = taurochenodeoxycholic acid, GUDCA = glycoursodeoxycholic acid, TDCA = taurodeoxycholic acid, G-conjugated = Glycine-conjugated bile acid, *T-conjugated* = Taurine-conjugated bile acid.

## Data Availability

The data presented in this study are available on request from the corresponding author. The data are not publicly available due to ethical reasons.
